# Neurocognitive performance of patients undergoing intravenous versus oral opioid agonist treatment: a prospective multicenter study on three-month treatment effects

**DOI:** 10.3389/fpsyt.2024.1375895

**Published:** 2024-07-23

**Authors:** Sunsha Chamakalayil, Rudolf Stohler, Andreas Moldovanyi, Markus Gerber, Serge Brand, Kenneth M. Dürsteler

**Affiliations:** ^1^ Faculty of Medicine, University of Basel, Basel, Switzerland; ^2^ Psychiatric Practice Aquila, Pratteln, Switzerland; ^3^ Division of Sport and Psychosocial Health, Department of Sport, Exercise, and Health, University of Basel, Basel, Switzerland; ^4^ Substance Abuse Prevention Research Center, Kermanshah University of Medical Sciences (KUMS), Kermanshah, Iran; ^5^ Sleep Disorders Research Center, Kermanshah University of Medical Sciences (KUMS), Kermanshah, Iran; ^6^ Center for Affective, Stress and Sleep Disorders, Psychiatric University Clinics Basel, University of Basel, Basel, Switzerland; ^7^ School of Medicine, Tehran University of Medical Sciences (TUMS), Tehran, Iran; ^8^ Center for Disaster Psychiatry and Disaster Psychology, Psychiatric University Clinics Basel (UPK), University of Basel, Basel, Switzerland; ^9^ Division of Substance Use Disorders, Psychiatric University Clinics Basel, Basel, Switzerland; ^10^ Department for Psychiatry, Psychotherapy and Psychosomatic, Psychiatric Hospital, University of Zurich, Zurich, Switzerland

**Keywords:** neurocognitive performance, opioid dependence, opioid agonist treatment, intravenous diacetylmorphine (IV-DAM), oral opioids

## Abstract

**Introduction:**

The first-line treatment for opioid dependence is opioid agonist treatment (OAT) with oral opioids. However, in some cases, treatment with intravenous diacetylmorphine (IV-DAM) is indicated. Research on neurocognitive impairments and treatment effects of OAT - particularly with IV-DAM - on neurocognitive functioning, is scarce. The current study is the first to investigate the neurocognitive performance of individuals on OAT with IV-DAM. Using a prospective study design with two timepoints of measurement, the first aim was to assess the nature and extent of neurocognitive functioning in individuals with opioid dependence by comparing participants’ neurocognitive performance with normative data of the general population on admission to treatment (baseline) and after an initial three-month period of OAT (study end). The second aim was to examine whether and to what extent neurocognitive performance would improve after three months on OAT. The third aim was to investigate whether, and if so, to what extent the treatment method (IV-DAM vs. oral opioids) would lead to higher neurocognitive improvements at study end.

**Methods:**

Forty-seven opioid-dependent individuals (baseline; 33 individuals at study end) participated in this study (mean age: 34.3 years; 27.7% female). Participants underwent neuropsychological testing with a battery of 12 tests covering different neurocognitive domains, including attention, memory, and executive functions.

**Results:**

Compared to normative data, opioid-dependent individuals showed impairments in almost every test both at baseline and at study end. At baseline, neurocognitive performance did not differ between individuals receiving IV-DAM or oral opioids for OAT. Compared to baseline, the neurocognitive performance did neither improve nor deteriorate after three months of treatment with neither IV-DAM nor oral opioids. However, a trend towards improvement was found for the memory domain.

**Discussion:**

Given that neurocognitive impairments should be considered in treatment planning and therapeutic interventions. Since a reduced cognitive performance may affect both the treatment outcome and the therapeutic relationship unfavorably, specific neurocognitive training at the beginning of treatment should be considered.

## 1 Introduction

Opioid dependence is a major public health concern worldwide (1). It is estimated that around 60.3 million (range: 37 -76 million) people had used opioids in 2021 (2), and the estimated number of opioid users worldwide has nearly doubled from 2010 to 2019 (3). In 2017, The Global Burden of Disease Study estimated that around 40.5 million people worldwide were dependent on opioids (4).

In opioid agonist treatment (OAT) with intravenous diacetylmorphine (IV-DAM) or oral opioids, a thorough and proper adherence to treatment conditions was associated with improved treatment outcomes (5–7). In contrast, individuals discontinuing their treatment (5, 6), or using illicit or non-prescribed pharmaceutical opioids additionally were at increased risk of falling back to pre-treatment levels of opioid use (5, 7). Therefore, identifying risk factors for these treatment outcomes is essential (8). More specifically, neurocognitive impairment is a significant risk factor for adverse treatment outcomes (8). It may significantly deteriorate daily life activities and treatment outcomes (9, 10). Impairments in learning, memory, and executive functions affect the ability to manage finances, holding down a job, and everyday tasks like driving, interacting in social situations and keeping appointments (11, 12). Additionally, these impairments might increase the risk of poor treatment outcomes, including premature treatment dropout (13, 14), low attendance at treatment sessions (15), low motivation to behavioral change (16), poor medication adherence (17), high impulsivity, and higher levels of substance use during treatment and after treatment termination (13, 18, 19). Further, poor treatment outcomes often lead to higher utilization of treatment services, e.g., in form of re-admissions to OAT. Therefore, the presence of neurocognitive impairments in patients undergoing OAT may result in higher costs (20). Additionally, the treatment of these patients requires more sophisticated strategies and thus more time and personnel resources (20). Consequently, identifying and addressing neurocognitive impairments appears crucial in improving treatment outcomes and the overall efficacy of OAT.

As regards the neurological dimension, long-term use of opioids has been associated with deteriorated structural and functional neuronal changes, which were accompanied by neurocognitive impairments (21–23). Specifically, long-term heroin use, was associated with impairments in frontal white matter bilaterally (21, 24–26), and with the reduction in frontotemporal grey matter density bilaterally (27–30). Also, changes in cortical and subcortical connectivity were identified after long-term heroin use (21, 31). Long-term parenteral (intravenous or intranasal) heroin use led to recurrent hypoxic events during non-fatal overdoses, which in turn were associated with brain lesions (32, 33). In treatment with IV-DAM, hypoxic events occur repeatedly (34, 35). Given such structural and functional alterations (23), neurocognitive impairments due to these hypoxic events are conceivable (29, 33, 34).

To determine the extent of neurocognitive functions of treated and untreated heroin users several studies employed neuropsychological tests, though the pattern of results appeared inconsistent (20). Some early studies found no effect of long-term use of street heroin on standard neuropsychological tests (36–38), whereas others reported neurocognitive impairments (39–42). Further, early investigations on neurocognitive effects of long-term heroin use showed that individuals with heroin dependence were more likely to show poor performance on traditional neuropsychological tests such as the Halstead-Reitan-Battery and the Wechsler Adult Intelligence Scale (39, 43, 44). Impairments were also observed for memory (41, 45), but relatively few impairments for tasks involving abstraction and reasoning (40, 41, 44, 46–50). However, studies using more sensitive neuropsychological tests have demonstrated that heroin-dependent individuals may also have substantial impairments in frontal lobe functioning, but that these impairments may not necessarily include issues of altered attentional control, altered decision making, or cognitive flexibility as measured by the Wisconsin Card Sorting Test (51–54). To illustrate, when compared to healthy controls, heroin-dependent individuals three weeks post-detoxification and with no significant history of other substance use showed impairments in general motor ability, short-term memory, problem solving, decision making, and cognitive flexibility, when compared to healthy controls (43). Recent systematic reviews suggest that in opioid-dependent individuals, impairments can be identified in several neuropsychological domains like attention, impulsivity, short-term memory, visuospatial ability, verbal fluency, executive functioning, and complex psychomotor domain, with small to large effects compared to norm population (42, 55–57).

Generally, more knowledge on the effectiveness of OAT on neurocognitive performance is needed. However, studies investigating the prevalence and severity of neurocognitive impairments among opioid-dependent individuals entering OAT, particularly IV-DAM treatment, are not extensively available. Further, it remains unclear whether these impairments may be reversible at least in parts under stable treatment conditions. Furthermore, it is not well investigated whether OAT, particularly with IV-DAM, affects the neurocognitive functioning of opioid-dependent individuals (58). On the one hand, in long-term methadone-treated individuals, impairments in impulsivity, cognitive flexibility, attention, and memory were reported (55). On the other hand, studies showed that OAT with oral opioids might improve neurocognitive functioning over time in the domains of verbal learning, visuospatial memory, processing speed, and executive functioning (58–63). These improvements were identified as early as two months after OAT initiation (60, 63). Also, improvements in learning and memory in association with medication adherence were determined in patients on OAT over six months; in contrast, such improvements were not observed for attention, short-term memory, processing speed, or executive functioning (58). Nevertheless, a recent systematic review suggests that compared to untreated opioid-dependent individuals, those on OAT performed better on attention, memory, and executive functioning (57). However, it appears that there are no studies, which investigated the effect of OAT with IV-DAM on neurocognitive functions of opioid-dependent patients.

In the same vein, there are no studies comparing the effect of OAT with IV-DAM versus oral opioids on neurocognitive functioning in patients with opioid dependence. Yet, the difference in the treatment effect on neurocognitive functioning between the two treatments might be an important factor to be considered, when choosing between the two treatment approaches.

In view of these gaps of research, the aims of the present study with a prospective design and two timepoints of measurement, were threefold: First, to compare the neurocognitive performance in opioid-dependent individuals both at baseline and at study end to normative data from the general population. Second, to assess these neurocognitive performances both at baseline and three months later after continuous OAT with either IV-DAM or oral opioids. Third, to investigate, whether, and if so, to what extent, the treatment approach (IV-DAM vs. oral opioids) would yield higher neurocognitive improvements at the end of the intervention three months later. First, following others (41, 42, 55, 56), we hypothesized that participants’ neurocognitive performance would be lower both at baseline and study end, when compared to normative data. Second, following others (58–63), we hypothesized that a comprehensive OAT with IV-DAM or oral opioids for three months would improve neurocognitive functioning. Third, based on previous research (29, 33, 34), we expected that the improvement would be lower on OAT with IV-DAM than with oral opioids.

## 2 Materials and methods

### 2.1 Participants

The baseline sample consisted of 47 outpatients with severe opioid dependence. Inclusion criteria were: age between 18 and 65 years; diagnosis of heroin dependence according to DSM-IV, as ascertained by trained and experienced psychiatrists; (Swiss) -German as the native language or as a second language beginning by the age of 7 years; willing and able to comply with the study conditions; new admission or readmission to OAT with IV-DAM or oral opioids; and signed written informed consent. Exclusion criteria were any current co-occurring severe mental health condition other than substance use disorder and any medical condition judged to be clinically significant for the integrity of neurocognitive functioning, and any history of severe cerebral trauma. Socio-demographic information (sex, age, years of heroin use, dose, and illness-related information) were taken from the electronic medical records.

### 2.2 Procedures and measures

#### 2.2.1 Procedures

All outpatients with opioid dependence and assigned to OAT with IV-DAM or oral opioids were approached to participate in the present study. Out of these patients, 47 agreed to take part. These individuals attended seven treatment centers located in Zürich (n = 5) and Basel (n = 2), Switzerland, during the period of January 2001 to December 2001. Participants were fully informed about the aims of the study and the confidential handling of sensitive data. They then signed a written informed consent. Next, they underwent neurocognitive testing both at baseline and 3 months later. The Cantonal Ethic Committee of Zurich approved the study, which was performed in accordance with the 6th Revision of the Declaration of Helsinki (64).

#### 2.2.2 Neuropsychological test sessions

Neuropsychological test sessions took place in separate, adequately lit rooms selected to provide a comfortable and quiet atmosphere with a consistent level of lighting, sound, heat, and visual stimuli. The time of testing was set individually but was intended to preclude, as much as possible, any disruptive effects of acute opioid administration or opioid withdrawal on neuropsychological test performance. Before the sessions experienced psychiatrists assessed whether participants showed signs of intoxication or withdrawal. During neuropsychological testings, participants were not allowed to smoke tobacco cigarettes, eat food, or drink stimulating beverages.

Before the baseline neuropsychological assessments, participants were interviewed by the examiner regarding their history of neurological events associated with poor functional outcomes and their current and past substance use. The interviews were based on a self-developed questionnaire (see **Supplementary Materials**) and on a short version of the Drug and Alcohol Section of the European Addiction Severity Index (65, 66). The short version of the Drug and Alcohol Section of the European Addiction Severity Index was also administered at study end. All neuropsychological data were collected in paper-and-pencil format.

The test sessions lasted approximately 2 hours, and participants were encouraged to take a 15-minute rest period halfway through the session. After the test sessions, patients were screened for the presence of opiates, benzodiazepines, cocaine, cannabis, and amphetamine in their urine.

#### 2.2.3 Neuropsychological measures

The neuropsychological battery consisted of tests with proven reliability and validity (67). It covered the major components of neurocognitive functioning, including premorbid verbal intelligence, attention, learning and memory, cognitive flexibility, motor coordination, and information processing. It was designed to measure selected functional domains that are potentially impaired in extreme-altitude climbers (68, 69). Since they may experience comparable levels of hypoxia as patients with heroin dependence (70), it was also used to measure potential hypoxia-related impairments in opioid-dependent individuals. To increase participant adherence, the entire battery was kept as short as possible with some modifications to the standard processing. The battery consisted of the following tests, most of which have been described by Lezak (67). The tests were administered in this standard order:

− Premorbid verbal intelligence: Mehrfachwahl-Wortschatz-Intelligenztest (MWT, multiple-choice vocabulary intelligence test) is a 37-item multiple-choice test assessing the level of vocabulary. This test was used as an estimate of premorbid intellectual functioning. A higher sum score indicates a higher level of vocabulary and estimated premorbid intellectual functioning.
− Visuo-graphomotor functioning and visual memory: Complex Figure Test (CFT). The copy task of the CFT examines the perceptual organization and graphomotor visuo-constructive skills, while the 60-minute delayed reproduction task assesses visuospatial memory. Participants were asked to copy a complex geometric figure (Rey Osterrieth Complex Figure) (71) at baseline and from the Taylor Figure Test (TFT) (67) at the study end. Sixty minutes after copying, participants were asked to reproduce the figure from memory without warning. A higher sum score indicates a higher level of perceptual organization, graphomotor visuo-constructive skills, and visuospatial memory.
− Verbal learning and memory: Rey Auditory Verbal Learning Test (RAVLT) (72) is a widely used measure of verbal learning, short-term, and longer-term verbal memory. A higher sum score indicates a higher level of verbal learning and memory.
− Visual learning and memory: Rey Visual Design Learning Test (RVDLT) (73) is a commonly used task assessing visual learning, short-term, and longer-term visual memory. A higher sum score indicates a higher level of visual learning and memory.
− Generative verbal fluency: S-Word Fluency Test (S-WFT) is commonly used as an index of executive verbal function. A higher sum score indicates a higher level of executive verbal function (74).
− Cognitive interference: Stroop Test (ST; color-word-interference trial; Victoria Version) (75, 76) is a test of executive functioning, which provides a measure of attentional set-shifting and cognitive interference caused by incongruity. A higher score of interference (Stroop C/Stroop A) indicates a higher level of interference.
− Figural response fluency: Five-Point Test (5PT) (77) explores figural executive functioning. A higher sum score indicates a higher level of figural executive functioning.
− Adaptive flexibility: Goldenberg Test (GT) assesses the function of adaptive flexibility in learning. A higher sum score indicates a higher level of adaptive flexibility in learning.
− Selective attention: d2-Test (78) measures processing speed, rule compliance, and quality of performance and allows for an estimation of selective visual attention and concentration. A higher sum score indicates a higher level of selective visual attention and concentration.
− Information processing and motor coordination: Digit Symbol Substitution Test (DSST) assesses general perceptual-graphomotor speed and accuracy as well as sustained attention and visual short-term memory. A higher sum score indicates a higher level of general perceptual-graphomotor speed, accuracy, sustained attention, and visual short-term memory.
− Auditory attention and short-term memory: The Wechsler Memory Scale – Forward Digit span (FDS) provides a measure of auditory attention and short-term memory while Backward Digit Span (BDS) backward measures short-term memory capacity and additionally test executive functioning (manipulation and cognitive control). A higher sum score indicates a higher level of auditory attention, short-term memory, and executive functioning.

Neuropsychological measures at study end after the three-month initial treatment period: Except for marginal modifications, the neuropsychological battery included the same tests as employed at baseline. The modifications were as follows: The MWT was not administered, the CFT was replaced by the TFT, word lists A and B of the RAVLT were replaced by word lists C and D.

#### 2.2.4 Subject-rated measures

To control for potential confounding factors due to psychological distress and depressive symptomatology, participants completed two self-assessment instruments the day before testing, the Beck Depression Inventory (BDI) (79) and the Symptom Check-List-90-R (SCL-90-R) (80).

### 2.3 Statistical analyses

All statistical computations were performed with SPSS^®^ 28.0 (IBM Corporation, Armonk, NY, USA) for Windows^®^. The level of significance was set at α < 0.05.

First, a series of Pearson’s correlations was performed to calculate the associations between age, depression, psychiatric symptoms, years of heroin use, days of heroin use in the last 30 days, dose (daily opioid doses are given in methadone equivalent doses using a conversion ratio of 1:4 for injectable diacetylmorphine and 1:8 for oral diacetylmorphine), and neurocognitive performances, both at baseline and three months later at study end. Where normal distribution was not given (dose at baseline), Spearman’s correlations were performed. Then, we examined whether and to what extent participants’ neurocognitive performances were equal or below standards. To this end, a series of one-sample t-tests of participants’ neurocognitive performances at baseline and study end with normative data was performed. We defined trivial small, medium, and large effect sizes based on Cohen’s d, categorizing values below 0.2 as indicative of a trivial effect, between 0.2 and 0.49 as a small effect, between 0.5 and 0.79 as indicative of a medium effect, and values above 0.8 as a large effect. Next, performance results of all tests were z-transformed.

Based on theoretical construct of the tests and the associations between the tests, neurocognitive tests were clustered into the following three dimensions: attention, memory, and executive functions. Neurocognitive tests were aggregated to indices, i.e., an index score for attention, memory, and executive functions. The index score was built by calculating the mean for each dimension using the z-scores of tests assigned to each dimension. These index scores were correlated once again with age, depression, and psychiatric symptoms, both at baseline and at study end. A series of t-tests for related samples were used to detect differences between both the test results and index scores of neurocognitive performances at baseline and study end.

Further, the neurocognitive performance of participants was clustered into five groups from clearly below average performance to clearly above average performance compared to norm population for baseline and study end to have a more detailed picture of the neurocognitive performance of the participants. Finally, to investigate the factors influencing the performance of the indices attention, memory, and executive functions at study end, a multi regression analyses was conducted with the variables performance at baseline, administration route (intravenous vs. oral), intelligence, age, sex, and psychiatric symptoms.

## 3 Results

### 3.1 General information on the sample

**Table 1** gives a descriptive statistical overview of sociodemographic and illness-related information, separately for participants treated with IV-DAM and oral opioids.

**Table 1:** Descriptive statistical overview of sociodemographic and illness-related information, separately for participants treated with IV-DAM (intravenous diacetylmorphine) and oral opioids.

Treatment conditions						
	IV-DAM		Oral opioids		Total	
	Baseline	Study end	Baseline	Study end	Baseline	Study end
N (n females)	25 (7f)	18 (6f)	22 (6f)	15 (5f)	47 (13f)	33 (11f)	
	Mean (SD)		Mean (SD)		Mean (SD)		
Dose	53.75 (19.45)	116.33 (22.79)	100.50 (75.21)	79.31 (105.01)	111.25 (71.61)	126.90 (79.46)	
BDI	15.92 (10.23)	13.06 (8.02)	11.36 (7.77)	12.80 (10.63)	13.74 (9.33)	12.94 (9.18)	
Psychiatric symptoms	62.30 (13.82)	62.07 (13.65)	58.94 (11.84)	58.50 (13.97)	60.76 (12.88)	60.48 (13.64)	
	IV-DAM		Oral opioids		Total		
Age in years	33.60 (5.94)		34.55 (8.71)		34.04 (7.30)		
Heroin use in years	13.02 (4.35)		8.43 (5.03)		10.83 (5.18)		

IV-DAM, Intravenous diacetylmorphine; f, female; SD, standard deviation; BDI, Beck’s Depression Inventory; Dose: Methadone equivalent dose in mg using conversion ratio of 1:4 for injectable diacetylmorphine and 1:8 for oral diacetylmorphine.

### 3.2 Correlations between age, depression, psychiatric symptoms, dose, years of heroin use, and neurocognitive performance at baseline and study end

**Supplementary Table S1** and **Supplementary Table S2** (see **Supplementary Materials**) show correlations between age, depression, psychiatric symptoms, dose, years of heroin use, and neurocognitive performance at baseline and study end. The correlations between the neurocognitive tests and theoretical constructs were taken into consideration while developing the three indices for attention, memory, and executive functions. CFT/TFT and ST hardly correlated with any other test; therefore, both tests were not considered for indices.

#### 3.2.1 Correlations at baseline

Higher age at baseline was significantly associated with more years of heroin use, higher verbal intelligence (MWT) and higher scores in S-WFT (executive functioning). For all other dimensions, correlation coefficients were trivial or not significant. Higher depression scores were significantly associated with higher psychiatric symptoms. Higher psychiatric symptoms were significantly associated with lower scores for S-WFT (executive functioning). More years of heroin use was significantly associated with more days of heroin use in the last 30 days and higher scores for S-WFT (executive functioning). Higher heroin use in the last 30 days was significantly associated with higher scores for d2-T (attention), FDS (attention) and S-WFT (executive functioning). Higher scores for MWT (verbal intelligence) were associated with higher scores for d2-T (attention), DSST (attention), RVDLT (memory) and S-WFT (executive functioning), 5PT (executive functioning) and GT (executive functioning).

#### 3.2.2 Correlations at study end

Higher age at study end was significantly associated with more years of heroin use, more heroin use in the last 30 days and higher verbal intelligence (MWT). For all other dimensions, correlation coefficients were trivial or not significant. Higher depression scores were significantly associated with higher psychiatric symptoms and lower verbal intelligence (MWT), lower scores for DSST (attention), RAVLT (memory), S-WFT (executive functioning), and BDS (executive functioning). Higher psychiatric symptoms were significantly associated with lower verbal intelligence (MWT), higher heroin use in the last 30 days, and lower scores for S-WFT (executive functioning). More years of heroin use was significantly associated higher heroin use in the last 30 days, with lower scores for RAVLT (memory) and with higher scores for S-WFT (executive functioning). Higher verbal intelligence (MWT) was significantly associated with higher scores in d2-T (attention), S-WFT (executive functioning), and BDS (executive functioning).

### 3.3 Correlations between age, depression, psychiatric symptoms, heroin use in years, heroin use in last 30 days, dose, intelligence, and neurocognitive performance (indices) both at baseline and study end

**Supplementary Table S3** and **Supplementary Table S4** (see **Supplementary Materials**) give an overview of correlation coefficients (Pearson’s and Spearman’s correlations) between age, depression, psychiatric symptoms, heroin use in years, heroin use in last 30 days, dose, intelligence, and neurocognitive performance (indices) at baseline and at study end.

#### 3.3.1 Correlations at baseline

Higher scores for attention were significantly associated with higher scores for heroin use in last 30 days, memory, executive functioning, and verbal intelligence. Higher memory scores were significantly associated with higher scores for executive functioning and verbal intelligence. Higher executive functioning scores were significantly associated with higher scores for heroin use in last 30 days and verbal intelligence.

#### 3.3.2 Correlations at study end

Higher scores for attention were significantly associated with lower scores for depression and psychiatric symptoms, higher scores for memory, executive functioning, and verbal intelligence. Higher scores for memory were significantly associated with lower scores for psychiatric symptoms. Higher executive functioning scores were significantly associated with higher scores for verbal intelligence. The correlation between memory and executive functioning was not significant.

### 3.4 Comparison between neurocognitive performance of participants and normative data

**Table 2** provides the descriptive and inferential statistical overview of participants’ neurocognitive performances at baseline and study end, compared to normative data. Participants’ neurocognitive performances both at baseline and study end were lower (mostly medium to large effect sizes), except for GT (executive functioning) at baseline and S-WFT (executive functioning) at study end. Small effect sizes were found for S-WFT (executive functioning) at baseline and GT (executive functioning), d2-T (attention), and DSST (attention) at study end. Overall, compared to normative data from healthy populations, participants’ neurocognitive performance was lower at baseline and at the end of the study.

**Table 2:** Overview of T-tests with baseline and study end with normative data.

Variables Baseline						
	Mean	SD	Mean (Norm. data)	T	df	Cohen’s d
Attention						
d2-T	401.47	78.70	462.40	-5.31	46	-.77***
DSST	48.87	11.50	57.30	-4.97	45	-.73***
FDS	7.65	2.08	6.7	3.12	45	.46**
Memory						
RAVLT	7.64	3.59	10.6	-5.66	46	-.83***
RVDLT	6.83	3.33	14.07	-14.73	45	-2.17***
RAVLT (L)	9.21	2.11	11.30	-6.79	46	-.99***
RAVLT (L)	6.28	2.24	11.52	-16.02	46	-2.34***
Executive functions						
5PT	27.64	6.980	37.70	-9.88	46	-1.44***
S-WFT	29.76	10.36	33.30	-2.75	46	-.40*
GT	54.45	8.96	56.20	-1.34	46	-.20
BDS	6.35	2.35	5.3	3.03	45	.45**
Intelligence						
MWT	29.17	3.178	26.5	5.76	46	3.178***
Variables Study End						
Attention						
d2-T	415.33	108.49	462.40	-2.49	32	-.43**
DSST	53.30	13.24	57.30	-1.74	32	-.30*
FDS	7.48	2.00	6.7	2.25	32	.39*
Memory						
RAVLT	7.58	3.00	10.60	-5.79	32	-.98***
RVDLT	7.73	2.79	14.07	-13.07	32	-2.28***
RAVLT (L)	9.35	1.76	11.30	-6.354	32	-1.11***
RVDLT (L)	6.70	2.07	11.52	-13.38	32	-2.33***
Executive functions						
5PT	33.09	6.49	37.70	-6.74	32	-1.17***
S-WFT	30.58	10.18	33.30	-1.54	32	-.27
GT	52.16	10.30	56.20	-2.21	32	-.39*
BDS	6.30	2.34	5.3	2.46	32	.43**

Cohen’s d ≥ 0.8 = large; 0.5 - 0.79 = medium; 0.2 - 0.49 = small; < 0.2 = trivial. *p ≤.05, ** p ≤.01, ***p ≤.001.

d2-T, d2-Test; DSST, Digit Symbol Substitution Test; FDS, Forward Digit Span; RAVLT (L), Rey Auditory Verbal Learning Test (long term); RVDLT (L), Rey Visual Design Learning Test (long term); 5PT, Five-Point Test; S-WFT, S-Word Fluency Test; GT, Goldenberg Test; BDS, Backward Digit Span.

### 3.5 Comparison between neurocognitive tests at baseline and study end

**Supplementary Table S5** (see **Supplementary Materials**) provides the descriptive and inferential statistical comparison of variables between baseline and study end. Significant attention improvements were observed for the DSST (medium effect size), while for other dimensions effect sizes were trivial. Significant memory improvements were found for RVDLT (L) (medium effect size). No mean differences were observed for RAVLT (trivial effect size). Significant executive functioning improvements were found for the 5PT (large effect size). For all other tests, effect sizes were trivial to small.

### 3.6 Statistical comparisons of index values at baseline and study end

**Table 3** provides the descriptive and inferential statistical overview of index values at baseline and study end. Changes in attention, memory, and executive functioning were trivial or small and statistically not significant.

**Table 3:** Overview of T-tests on neurocognitive performance between baseline and study end.

Timepoints							
	Baseline		Study end				
	Mean	SD	Mean	SD	T	df	Cohen’s d
Attention	-.02	1.03	.05	.96	-.32	31	-.06
Memory	-.05	.99	-.02	1.01	-.30	31	-.05
Executive functions	.00	.97	.09	.88	-.86	31	-. 15

Cohen’s d ≥ 0.8 = large; 0.5 - 0.79 = medium; 0.2 - 0.49 = small; < 0.2 = trivial (t).

### 3.7 Classification of the neurocognitive performance for the indices at baseline and study end

**Table 4** provides the percentage of patients in each group classified according to the neurocognitive performance at baseline and study end.

**Table 4:** Classification of the neurocognitive performance in percentage for the indices at baseline and study end.

Variables					
	Clearly below average (Z-score = ≤ -1.6)	Below average (Z-score = > -1.6 - < -1.0)	Average (Z-score = ≥ -1.0 - ≤ 1.0)	Above average (Z-score = > 1.0 - < 1.6)	Clearly above average (Z-score= > 1.6)
Attention					
Baseline	6.5	6.5	73.9	8.7	4.3
Study end	6.1	9.1	69.7	9.1	6.1
Memory					
Baseline	6.4	10.6	72.3	6.4	4.3
Study end	3.0	9.1	78.8	3.0	6.1
Executive functions					
Baseline	6.4	8.5	66.0	14.9	4.3
Study end	6.3	6.3	71.9	12.5	3.1

At baseline, 13% of the participants had a below average performance, 74% an average performance and 13% an above average performance on tests on attention. At study end, 15% of the participants had a below average performance, 70% an average performance and 15% an above average performance on tests of attention.

At baseline, 17% of the participants had a below average performance, 72% an average performance and 11% an above average performance on tests on memory. At study end, 12% of the participants had a below average performance, 79% an average performance and 9% an above average performance on tests of memory.

At baseline, 15% of the participants had a below average performance, 66% an average performance and 19.2% an above average performance on tests on executive functions. At study end, 13% of the participants had a below average performance, 72% an average performance and 16% an above average performance on tests of executive functions.

### 3.8 Comparison of predictors of neurocognitive performance at study end

**Table 5** provides an overview of the predictors of neurocognitive performances (index values) at study end.

**Table 5:** Coefficients of the Multiple Linear Regression Model on Attention, Memory, and Executive Functions at Study End.

Predictor Variable	Coefficient		
	Attention	Memory	Executive functions
Baseline	.54*	.68*	.71*
Administration route: intravenous vs. oral	-.00	.39	.09
Intelligence	.14	-.02	.14
Age	-.01	.00	-.01
Sex	.13	-.02	.29
Psychiatric Symptoms	-.27	-.17	.10

*p <.001.

The multiple linear regression model accounted for 67% of the variance in attention performance at follow-up (Adj. R-squared=.67). The regression model was statistically significant (F (5,13) =9.89, p= <.001). The coefficient for attention at baseline was significant (β=.62, p= <.001), indicating that higher attention performance at baseline was associated with higher attention performance at follow-up. No other coefficients were significant.

The multiple linear regression model accounted for 57% of the variance in memory performance at follow-up (Adj. R-squared=.57). The regression model was statistically significant (F (5,13) =6.83, p=<.001). The coefficient for memory at baseline was significant ((β=.68, p= <.001), indicating that higher memory performance at baseline was associated with higher memory performance at follow-up. No other coefficients were significant.

The multiple linear regression model accounted for 65% of the variance in executive function performance at follow-up (Adj. R-squared=.65). The regression model was statistically significant (F (5,13) =8.89, p=<.001). The coefficient for executive function at baseline was significant ((β=.77, p= <.001), indicating that higher executive function performance at baseline was associated with higher executive function performance at follow-up. No other coefficients were significant.

## 4 Discussion

This study explored whether the neurocognitive performance of opioid-dependent patients on OAT was comparable to the performance of the norm population. Further, the study investigated whether neurocognitive performance of opioid-dependent patients undergoing OAT improved after a three-month comprehensive treatment program. We also investigated if these improvements were lower with IV-DAM than with oral opioids.

The key findings of the present study were that compared to normative data, participants’ neurocognitive performance was poor both at baseline and at study end (mostly medium to large effect sizes). Further, over time and irrespective of the treatment condition (OAT with IV-DAM vs. oral opioids), descriptively, improvements on neurocognitive performances could be observed, though they were not significant and of trivial to small magnitude. In general, age, depressive symptoms and psychiatric symptoms were at maximum modestly related to neurocognitive performances.

With the first hypothesis, we assumed that compared to normative data of healthy populations participants’ neurocognitive performances would be lower at baseline and study end, and data did confirm this. As shown in **Table 2**, medium to large effect sizes were detected for all tests of attention and memory and for the 5PT and BDS of executive functioning at baseline. The present results are consistent with other studies (41–43, 45, 55–57). In our opinion, the present data confirm that opioid-dependent individuals have impairments in domains of attention, memory, and executive functioning. The findings of our study thus corroborate previous results in the field.

With the second hypothesis, we assumed that among opioid-dependent individuals, a comprehensive treatment program (OAT with IV-DAM or oral opioids) of three months would improve neurocognitive functioning and there would be some difference in the performance between the two treatment groups. Our data did only partially confirm these assumptions: As shown in **Supplementary Table S3**, improvement after three months of treatment with medium effect sizes were observed for RVDLT (L) (visual long-term memory), and the DSST (attention), and large effect sizes were observed for the 5PT (executive functioning). However, as displayed in **Table 3** and **Table 5**, overall, in all three domains, attention, memory, and executive functioning, there was no significant improvement after three months on OAT with either IV-DAM or oral opioids. Nevertheless, as shown in **Table 4**, there was a trend towards improvement in the memory domain after the three-month treatment period. Overall, the present results only partially corroborate the improvements observed elsewhere (57, 59–63). To our knowledge, the present data show for the first time that a three-month period of comprehensive OAT with IV-DAM does not differ from a three-month period of comprehensive OAT with oral opioids in terms of alterations in neurocognitive performance (**Table 5**).

Overall, the present results expand upon previous findings in four ways: First, three months after specific opioid dependence treatment, neurocognitive performance remained low, and improvements were modest. Second, there was no significant difference in the neurocognitive performances of opioid-dependent patients receiving three months OAT with IV-DAM or oral opioids, i.e., neurocognitive performance was unrelated to treatment condition (IV-DAM or oral opioids). Third, neurocognitive performance was modestly related to mental health symptoms in general and depressive symptoms in specific. Fourth, we thoroughly assessed neurocognitive domains broadly covering attention, memory, and executive functions.

For several reasons, we hold that the present results are of clinical and practical importance: First, given that compared to normative data from healthy populations, neurocognitive performances remained lower both at baseline and at study end, these results might suggest that psychosocial interventions in individuals with opioid dependence should consider participants’ neurocognitive capacities. Second, complementarily, these results might also suggest forming psychosocial interventions on a behavioral and experiential level rather than on a cognitive level, at least during the initial treatment period. Third, when treating and interacting with opioid-dependent patients, staff members might routinely double-check whether and to what extent patients on OAT are able to elaborate the meaning of the interventions. Fourth, since these results suggest that OAT for three months, which also implies stabilization of the opioid use and reduction of their drug-related lifestyle, is not sufficient to improve patients’ neurocognitive performance, the use of specific neurocognitive training batteries or apps seems required and might favorably impact patients’ neurocognitive performances as well as the use of opioids (9). Fifth, in terms of treatment effects on neurocognitive performance, OAT on IV-DAM is a comparable option to OAT with oral opioids, e.g., when deciding between both OAT approaches.

Despite the novelty of the present results, the following limitations should be considered. First, the sample size might appear small, although we focused on effect size calculations, which are not sensitive to sample size. Second, for reasons of statistical power, we did not investigate possible sex differences. This means that we do not know whether female compared to male participants showed similar or dissimilar neurocognitive performances. Third, while we thoroughly assessed participants’ neurocognitive functions of attention, memory, and executive functions participants’ capacities of decision-making, language, implicit information processing and practical problem solving were tested only to a limited extent. As such, participants’ neurocognitive performance in the present study predicts their skills to solve problems at the level of everyday life only to a certain degree. Fourth, it is conceivable that further latent but unassessed dimensions such as emotion regulation, impulsivity, and psychiatric disorders (e.g., ADHD) might have biased two or more dimensions in the same or opposite direction. Fifth, measuring the neurocognitive functioning after a treatment period of 12 or more months is important since it would allow to assess the effect of OAT in the development of neurocognitive performance over a longer period. It would also allow to better understand whether and to what extent the present neurocognitive performance could predict individuals’ illness-related information and risk behavior.

## 5 Conclusions

The results of this study may contribute to complete the profile of neuropsychological strengths and weaknesses of individuals with opioid dependence. In addition, it might bring new insights into the effects of OAT, particularly with IV-DAM, on neurocognitive functioning. We claim that the present results might be of practical and clinical importance because the findings might help to design specific neurocognitive treatment options for opioid-dependent patients. If so, this would also help opioid-dependent patients to cope with their specific neurocognitive impairments in their daily life.

## Data availability statement

The raw data supporting the conclusions of this article will be made available by the authors, without undue reservation.

## Ethics statement

The study was conducted in accordance with the Declaration of Helsinki and approved by the Cantonal Ethic Committee Zurich (E-037/2000; 2.11.2000).” for studies involving humans. 

## Author contributions

SC: Formal analysis, Visualization, Writing – original draft, Writing – review & editing. RS: Conceptualization, Data curation, Funding acquisition, Methodology, Project administration, Resources, Writing – original draft, Writing – review & editing. AM: Conceptualization, Data curation, Funding acquisition, Project administration, Writing – original draft, Writing – review & editing. MG: Resources, Writing – original draft, Writing – review & editing. SB: Formal analysis, Supervision, Writing – original draft, Writing – review & editing. KD: Conceptualization, Data curation, Funding acquisition, Methodology, Project administration, Resources, Supervision, Validation, Writing – original draft, Writing – review & editing.
