# Supplementary material for: Neurocognitive performance of patients undergoing intravenous versus oral opioid agonist treatment: a prospective multicenter study on three-month treatment effects
Source: Front Psychiatry. 2024 Jul 23;15:1375895. doi: 10.3389/fpsyt.2024.1375895 (PMC11300233; doi:10.3389/fpsyt.2024.1375895)
Supplement: Supplementary file 1 [file DataSheet_1.pdf]

Supplementary Material

Table S1.

Overview of Correlation Coefficients (Pearson's & Spearman's Correlations) Between Age, Depression, Psychiatric Symptoms, Heroin Use in Years, Heroin Use in Last 30 days, Opioid Dose, Intelligence and Neurocognitive Performance at Baseline.

								**Attention**	**Attention**	**Attention**
**Variables Baseline**	**Age**	**Depression**	**Psychiatric symptoms**	**Heroin use in years**	**Heroin use last 30 d**	**Dose**	**MWT**	**D2**	**DSST**	**FDS**
Age	–	-.06	-.10	.32*	.02	.01	.52*	.04	.02	.25
Depression		–	.69*	-.02	-.20	.26	-.18	-.15	-.21	-.21
Psychiatric symptoms			–	.01	-.22	.50	-.21	-.04	-.19	-.14
Heroin use in years	.			–	.44*	-.08	.08	-.05	-.08	.29
Heroin use last 30d					–	.33	.18	.35*	.29	.33*
Dose							.09	.09	.29	.29
MWT							–	.44*	.49*	.40*
**Attention**										
D2								–	.62*	.33*
DSST									–	.42*
FDS										–

	Memory	Memory	Memory	Memory	Memory	Executive functions	Executive functions	Executive functions	Executive functions	Executive functions
**Variables Baseline**	**CFT**	**RAVLT**	**RVDLT**	**RAVLT (L)**	**RVDLT (L)**	**S-WFT**	**ST**	**5PT**	**GT**	**BDS**
Age	-.10	-.18	.06	-.15	-.12	.29*	.03	.07	-.07	.24
Depression	.16	-.03	.08	.15	.01	-.24	.05	.02	-.13	-.13
Psychiatric symptoms	.04	.02	-.04	.16	-.07	-.20*	.02	-.01	-.13	-.06
Heroin use in years	-.08	-.14	.00	-.30	.00	.31*	-.10	.05	.05	.12
Heroin use last 30d	-.07	-.09	.18	-.31	.16	.35*	.00	.21	.23	.17
Dose	.28	.18	.46	.03	.46	.20	.01	.15	.03	.22
MWT	.20	.23	.35*	.09	.25	.44*	.10	.52*	.16	.50*
**Attention**										
D2	.13	.19	.29*	.12	.25	.41*	.17	.53*	.31*	.13
DSST	.11	.59*	.45*	.44*	.48*	.47*	.19	.46*	.42*	.48*
FDS	.22	.34*	.39*	.13	.55*	.68*	-.27	.32*	.42*	.57*
**Memory**										
CFT	–	.15	.17	.11	.22	.14	.12	.36*	.17	
RAVLT		–	.46*	.78*	.73*	.31*	.21	.24	.48*	.43*
RVDLT			–	.38*	.79*	.22	.07	.11	.10	.35*
RAVLT (L)				–	.58*	.06	.29*	.14	.31*	.20
RVDLT (L)					–	.31*	.22	.22	.49*	.45*
**Executive functioning**										
S-WFT						–	.05	.30*	.36*	.55*
ST							–	.11	.40	-.45*
5PT								–	.40*	.29
GT									–	.28
BDS										–

Note. MWT: Mehrfachwahl-Wortschatz-Intelligenztest, d2-T: d2-Test, DSST: Digit Symbol Substitution Test, FDS: Forward Digit Span, TFT: Taylor Figure Test, RAVLT (L): Rey Auditory Verbal Learning Test (long term), RVDLT (L): Rey Visual Design Learning Test (long term), S-WFT: S-Word Fluency Test, ST: Stroop Test, 5PT: Five-Point Test, GT: Goldenberg Test, BDS: Backward Digit Span.*p < .05

Table S2.

Overview of Correlation Coefficients (Pearson's Correlations) Age, Depression, Psychiatric Symptoms, Heroin Use in Years, Heroin Use in Last 30 days, Opioid Dose, Intelligence and Neurocognitive Performance at Study End.

							Intelligence	Attention	Attention	Attention
**Variables Study End**	**Age**	**Depression**	**Psychiatric symptoms**	**Heroin use in years**	**Heroin use last 30 d**	**Dose**	**MWT**	**d2-T**	**DSST**	**FDS**
Age	–	-.12	.10	.32*	.51*	.33	.52*	-.00	-.20	-.03
Depression		–	.59*	.07	.21	-.25	-.41*	-.32	-.59*	-.30
Psychiatric symptoms			–	.00	.49*	-.31	-.40*	-.30	-.50	-.58*
Heroin use in years				–	.54*	.55	.08	.05	.02	.22
Heroin use last 30 d					–	.19	.19	.14	-.27	-.18
Dose						–	.17	.10	.14	.05
MWT							–	.45*	.32	.33
**Attention**										
d2-T								–	.36*	.30
DSST									–	.51*
FDS										–

	Memory	Memory	Memory	Memory	Memory	Executive functions	Executive functions	Executive functions	Executive functions	Executive functions
**Variables Study End**	**TFT**	**RAVLT**	**RVDLT**	**RAVLT (L)**	**RVDLT (L)**	**S-WFT**	**ST**	**5PT**	**GT**	**BDS**
Age	.03	-.14	-.05	-.00	-.02	.26	.11	-.16	-.13	.06
Depression	-.34	-.38*	-.30	-.08	-.30	-.38*	-.00	-.31	.07	-.44*
Psychiatric symptoms	-.10	-.31	-.54*	-.10	-.37	-.19	-.15	-.08	-.13	-.33
Heroin use in years	-.20	-.26	-.01	-.35*	-.01	.45*	-.14	.04	.02	.24
Heroin use last 30 d	-.10	-.26	-.30	-.16	-.30	-.38	-.12	.17	.11	.17
Dose	.03	.18	.08	-.10	.26	.14	-.35	.09	.19	.07
MWT	.30	.21	.24	.12	.14	.49*	.21	.21	.19	.51*
**Attention**										
d2-T	-.05	.20	.32	.16	.17	.46*	-.10	.44*	.48*	.48*
DSST	.20	.45*	.37*	.38*	.20	.59*	.08	.54*	.12	.43*
FDS	.03	.17	.40*	.08	.13	.61*	-.46*	.42*	.42*	.69*
**Memory**										
TFT	--	.29	.22	.17	.37*	.05	.04	.24	.23	.14
RAVLT		--	.61*	.59*	.61*	.15	.24	.02	.13	.37*
RVDLT			--	.46*	.82*	.26	.26	.05	.33	.50*
RAVLT (L)				--	.44*	.14	.40*	.04	.17	.28
RVDLT (L)					--	.06	.16	-.10	.22	.36*
**Executive functions**										
S-WFT						--	-.07	.46*	.21	.57*
ST							--	-.15	.05	-.32
5PT								--	.46*	.36*
GT									--	.50*
BDS										--

Note. MWT: Mehrfachwahl-Wortschatz-Intelligenztest, d2-T: d2-Test, DSST: Digit Symbol Substitution Test, FDS: Forward Digit Span, TFT: Taylor Figure Test, RAVLT (L): Rey Auditory Verbal Learning Test (long term), RVDLT (L): Rey Visual Design Learning Test (long term), S-WFT: S-Word Fluency Test, ST: Stroop Test, 5PT: Five-Point Test, GT: Goldenberg Test, BDS: Backward Digit Span.*p < .05

Table S3.

Overview of Correlation Coefficients (Pearson's and Spearman's [With Dose-Baseline] Correlations) Between Age, Psychiatric Symptoms, Heroin Use in Years, Heroin Use in Last 30 Days, Dose, Intelligence and Neurocognitive Performance (Indices) at Baseline.

Variables Baseline	Age	Depression	Psychiatric Symptoms	Heroin use in years	Heroin use last 30 d	Dose	Attention	Memory	Executive functions	Intelligence
Age	—	-.06	-.10	.32*	.02	-.03	.15	-.12	.26	.52*
Depression		—	.69*	-.02	-.20	.47	-.25	.06	-.20	-.18
Psychiatric Symptoms			—	.01	-.22	.60	-.15	.02	-.13	-.21
Heroin use in years				—	.44*	-.59	.06	-.13	.17	.08
Heroin use last 30 d					—	-.34	.39*	.02	.31*	.18
Dose						—	.16	.24	.09	.09
Attention							—	.52*	.76*	.57*
Memory								—	.46*	.27
Executive functions									—	.63*
Intelligence										—

Note. *p < .05

Table S4.

Overview of Correlation Coefficients (Pearson's Correlations) Between Age, Psychiatric Symptoms, Heroin Use in Years, Heroin Use in Last 30 Days, Dose, Intelligence and Neurocognitive Performance (Indices) at Study End.

Variables Study end	Age	Depression	Psychiatric Symptoms	Heroin use in years	Heroin use last 30 d	Dose	Attention	Memory	Executive functions	Intelligence
Age	—	-.12	.10	.32*	.51*	.51*	-.10	-.06	.10	.52*
Depression		—	.59*	.07	.21	.15	-.52*	-.32	-.36	-.41*
Psychiatric symptoms			—	.00	.49*	.08	-.59*	-.39*	-.26	-.40
Heroin use in years				—	.54*	.10	.12	-.19	.24	.08
Heroin use last 30 d					—	.19	-.13	-.32	.31	.19
Dose						—	.14	.12	.28	.17
Attention							—	.39*	.81*	.52*
Memory								—	.32	.21
Executive functions									—	.48*
Intelligence										—

Note. *p < .05

Table S5.

Overview of T-Tests of Each Index Between Baseline and Study End

Variables	Mean (Baseline)	SD (Baseline)	Mean (Study end)	SD (Study end)	T	df	Cohen's d
**Attention**							
d2-T	437.12	92.29	436.18	114.87	.06	32	.02
DSST	49.41	11.88	53.78	13.16	-2.98	31	-.53*
FDS	7.63	1.88	7.59	1.93	.10	31	.02
**Memory**							
RAVLT	7.18	2.23	6.73	2.47	1.21	32	.21
RVDLT	4.39	2.02	4.82	1.94	-1.29	32	-.56
RAVLT (L)	7.85	3.40	7.58	3.00	.54	32	.09
RVDLT (L)	6.44	3.28	7.63	2.77	-2.91	31	-.52*
**Executive functions**							
S-WFT	29.76	10.36	30.58	10.18	-.65	32	.05
5PT	26.79	7.49	30.09	6.49	-3.14	32	-.91*
GT	14.35	1.11	14.29	.97	.28	30	.01
BDS	6.28	2.36	6.38	2.34	-.34	31	-.06

Cohen's d ≥ 0.8 = large; 0.5 - 0.79 = medium; 0.2 - 0.49 = small; > 0.2 = trivial. *p ≤ .05

Note. d2-T: d2-Test, DSST: Digit Symbol Substitution Test, FDS: Forward Digit Span, RAVLT (L): Rey Auditory Verbal Learning Test (long term), RVDLT (L): Rey Visual Design Learning Test (long term), S-WFT: S-Word Fluency Test, 5PT: Five-Point Test, GT: Goldenberg Test, BDS: Backward Digit Span.
